# Prognostic Impact of Pre-Treatment Modified Glasgow Prognostic Score (mGPS) on Survival in Patients with Advanced-Stage Ovarian Cancer

**DOI:** 10.3390/jcm14124239

**Published:** 2025-06-14

**Authors:** Fatih Kus, Firat Sirvan, Hasan Cagri Yildirim, Ilgin Koc, Naciye Guduk, Zafer Arik

**Affiliations:** 1 Department of Medical Oncology, Faculty of Medicine, Hacettepe University, 06000 Ankara, Turkey; 2 Department of Internal Medicine, Faculty of Medicine, Hacettepe University, 06000 Ankara, Turkey

**Keywords:** ovarian cancer, Glasgow prognostic score, prognosis

## Abstract

**Background:** Advanced-stage epithelial ovarian cancer is associated with variable survival outcomes, despite standardized treatments. Identifying reliable and accessible prognostic markers is critical to guide clinical decision-making. **Objective**: The aim of this study was to evaluate the prognostic significance of the modified Glasgow Prognostic Score (mGPS) in patients with FIGO stage III–IV epithelial ovarian cancer. **Methods**: In this retrospective cohort study, 89 patients diagnosed between 2018 and 2023 were analyzed. The mGPS was calculated from pre-treatment serum C-reactive protein (CRP) and albumin levels. Overall survival (OS) was assessed using Kaplan–Meier and Cox regression analyses. **Results**: The median OS was 32.3 months. When stratified by mGPS categories, the 2-year survival rates were 94%, 75%, and 34% in the mGPS 0, 1, and 2 groups, respectively (*p* < 0.001). In the multivariate Cox proportional hazards model, both mGPS (HR = 1.85; 95% CI: 1.12–3.07; *p* = 0.016) and ECOG performance status (HR = 1.67; 95% CI: 1.02–2.75; *p* = 0.043) were identified as independent predictors of overall survival. **Conclusions**: The mGPS is a simple, low-cost, and independently predictive tool for overall survival in advanced ovarian cancer. By capturing both systemic inflammation and nutritional status, it enhances risk stratification and may support individualized treatment planning. Prospective validation is warranted.

## 1. Introduction

Ovarian cancer remains one of the most lethal gynecological malignancies worldwide, mainly because it is often diagnosed at an advanced stage, limiting treatment options [1]. The current standard treatment consists of optimal cytoreductive surgery followed by platinum-based chemotherapy [2,3]. However, despite standardized treatment protocols, considerable variability in survival outcomes is observed among patients with similar disease stages receiving similar therapies [4]. This highlights the importance of understanding the biological factors that influence prognosis and identifying new prognostic markers that can be practically applied in clinical settings [5].

At the same time, treatment options for ovarian cancer have also improved in recent years [6]. Newer therapies such as PARP inhibitors, anti-angiogenic drugs like bevacizumab, and immune checkpoint inhibitors have started to play an important role, especially in patients with recurrent or platinum-sensitive disease [6,7]. For example, PARP inhibitors are now used as maintenance therapy in patients with BRCA mutations or other genetic changes that affect DNA repair [8].

In this context, there is an increasing demand for prognostic markers that are easily accessible, cost-effective, and reliable. The modified Glasgow Prognostic Score (mGPS) has emerged as a widely used biomarker that evaluates both systemic inflammation and nutritional status [9,10]. The mGPS is calculated based on serum C-reactive protein (CRP) and albumin levels. CRP reflects tumor-associated inflammatory activity and interleukin-6 levels, while serum albumin serves not only as an indicator of nutritional status but also as a surrogate marker of catabolic stress and physiological reserve [11]. By combining these two parameters, mGPS offers a comprehensive picture of both disease burden and the host’s response.

Numerous studies have demonstrated the prognostic utility of mGPS in various solid tumors, including gastrointestinal, lung, bladder, and endometrial cancers, showing a significant correlation with overall survival (OS) and progression-free survival (PFS) [12,13,14,15]. In ovarian cancer, systematic reviews and meta-analyses have similarly revealed an inverse association between high mGPS scores and both OS and PFS [9]. These findings suggest that mGPS may serve as a valuable prognostic tool in guiding clinical decision-making.

In addition to baseline biomarkers, dynamic markers reflecting treatment response have gained attention for their prognostic relevance. In recent years, dynamic biomarkers like the KELIM score, which measures the rate of CA-125 decline during chemotherapy, have also emerged as early indicators of treatment response. A 2023 multicenter study reported that the KELIM score was independently associated with OS and PFS in patients with platinum-resistant or refractory ovarian cancer [16]. This highlights the importance of incorporating kinetic parameters, alongside inflammation-based indices, into the clinical evaluation of ovarian cancer.

In this study, we retrospectively analyzed the impact of pre-treatment mGPS on overall survival in patients with advanced-stage epithelial ovarian cancer. The objective was to evaluate the clinical utility of the mGPS and to provide real-world evidence supporting its prognostic value in this patient population.

## 2. Materials and Methods

This retrospective cohort study was conducted at Hacettepe University Hospital, a tertiary care academic center in Turkey, and included patients treated between January 2018 and December 2023. The institutional ethics committee approved the study protocol, and all procedures were conducted in accordance with the Declaration of Helsinki.

### 2.1. Inclusion and Exclusion Criteria

Eligible participants were women aged ≥18 years with histologically confirmed advanced-stage epithelial ovarian cancer (International Federation of Gynecology and Obstetrics [FIGO] stages III–IV). All patients underwent either primary or interval cytoreductive surgery followed by platinum-based chemotherapy (carboplatin and paclitaxel). Patients were required to have documented pre-treatment serum C-reactive protein (CRP) and albumin levels measured within 10 days before initiating treatment.

The exclusion criteria included the following:

- The presence of synchronous malignancies;
- Evidence of acute infection or inflammatory conditions at the time of laboratory testing;
- Incomplete clinical or laboratory data;
- Receipt of neoadjuvant or experimental therapies outside the standard protocol.

### 2.2. Data Collection

Demographic data (age and ECOG performance status), clinical parameters (FIGO stage, histologic subtype, and tumor grade), laboratory results (CRP, albumin, and LDH), and treatment-related information were extracted from the institutional electronic medical record system. CRP and albumin were measured using standard automated immunoturbidimetric assays in a certified clinical laboratory.

### 2.3. mGPS Calculation

The modified Glasgow Prognostic Score (mGPS) was determined as follows:

- mGPS 0: CRP ≤ 10 mg/L and albumin ≥ 35 g/L;
- mGPS 1: CRP > 10 mg/L and albumin ≥ 35 g/L;
- mGPS 2: CRP > 10 mg/L and albumin < 35 g/L.

Patients with CRP ≤ 10 mg/L were assigned mGPS 0 regardless of albumin level, which is in line with validated scoring systems [17].

### 2.4. Outcome Measures and Statistical Analysis

The primary outcome was overall survival (OS), which is defined as the interval between the date of diagnosis and the date of death or the last follow-up. Survival outcomes were assessed using Kaplan–Meier estimates and compared using the log-rank test. Cox proportional hazards regression analysis was used to identify independent predictors of OS, adjusting for clinically relevant covariates (mGPS, ECOG performance status, age, and LDH level). All statistical analyses were performed using IBM SPSS Statistics version 25.0 (IBM Corp., Armonk, NY, USA). A two-sided *p*-value < 0.05 was considered statistically significant.

## 3. Results

The median age of the study population was 59 years (range, 25–84). At diagnosis, 75% of patients (*n* = 50) had an Eastern Cooperative Oncology Group (ECOG) performance status of 0–1, while 25% (*n* = 17) had a performance status of ≥2. According to FIGO staging, 68.8% of patients (*n* = 46) were classified as stage III and 31.2% (*n* = 21) were classified as stage IV (shown in Table 1).

The mean serum albumin level at diagnosis was 3.6 ± 0.65 g/dL, and the mean CRP level was 21.86 ± 20.47 mg/L. Based on the modified Glasgow Prognostic Score (mGPS), 19.1% (*n* = 17) of patients were classified as mGPS 0, 55.1% (*n* = 49) as mGPS 1, and 25.8% (*n* = 23) as mGPS 2.

**Figure 1:**
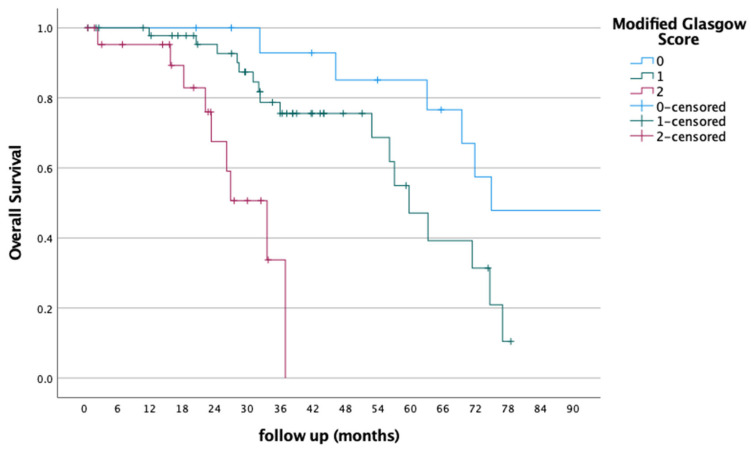
Kaplan–Meier overall survival curves stratified by mGPS score.

The median overall survival (OS) for the entire cohort was 32.3 months (range: 0.57–179.0). When stratified by mGPS categories, the 2-year survival rates were 94%, 75%, and 34% in the mGPS 0, 1, and 2 groups, respectively (*p* < 0.001) (shown in Figure 1)

In the multivariate Cox proportional hazards model, both mGPS (HR = 1.85; 95% CI: 1.12–3.07; *p* = 0.016) and ECOG performance status (HR = 1.67; 95% CI: 1.02–2.75; *p* = 0.043) were identified as independent predictors of overall survival. Serum lactate dehydrogenase (LDH) did not reach statistical significance (HR = 1.42; 95% CI: 0.97–2.09; *p* = 0.070). When analyzed as continuous variables, neither C-reactive protein (CRP) (HR = 1.11; 95% CI: 0.85–1.45; *p* = 0.352) nor serum albumin (HR = 0.88; 95% CI: 0.69–1.11; *p* = 0.276) was significantly associated with overall survival.

## 4. Discussion

This study assessed the prognostic significance of the modified Glasgow Prognostic Score (mGPS) prior to systemic therapy in patients with advanced-stage epithelial ovarian cancer. The results clearly demonstrate that higher mGPS scores are independently associated with shorter overall survival (OS). Patients with an mGPS of 0 had a median OS of approximately 75 months, while those with a score of 2 had a median OS of only 33.6 months. Similarly, 3-year survival rates declined markedly from 80% to 30% across increasing mGPS categories. These findings underscore the strong prognostic stratification capacity of mGPS and support its clinical relevance. The mGPS is derived from serum CRP and albumin levels, two widely available and inexpensive laboratory markers [18]. Elevated CRP levels reflect tumor-associated inflammatory responses, often mediated by interleukin-6 and other cytokines, while hypoalbuminemia indicates nutritional compromise, catabolic stress, and chronic systemic inflammation [14].

Beyond its clinical utility, the prognostic relevance of mGPS may be further explained through its underlying molecular associations. Chronic systemic inflammation, as indicated by elevated CRP, plays a crucial role in shaping the tumor microenvironment by promoting angiogenesis, epithelial–mesenchymal transition (EMT), and metastatic spread [19]. At the same time, inflammation can activate immune checkpoint pathways such as PD-1/PD-L1, leading to tumor immune escape [20]. These biologic mechanisms may partly explain why patients with elevated mGPS scores tend to exhibit more aggressive disease biology and poorer survival outcomes.

Moreover, malnutrition and catabolic stress—reflected by hypoalbuminemia—can lead to sarcopenia and treatment intolerance, both of which are associated with adverse oncologic outcomes [21]. Thus, mGPS may also indirectly reflect a patient’s functional reserve and treatment capacity. In future practice, mGPS could be utilized to guide prehabilitation strategies or nutritional interventions prior to initiating chemotherapy. Additionally, integrating mGPS into artificial intelligence-based predictive models may allow clinicians to generate individualized prognostic scores by combining laboratory, clinical, and radiologic data in a more dynamic manner.

In addition to mGPS, other simple blood-based scores have also gained attention in cancer care. One of them is the Prognostic Nutritional Index (PNI), which gives an idea of a patient’s immune system and nutritional health [22]. Research has shown that PNI is linked to survival in different types of cancer [23]. Just like mGPS, it reflects inflammation and poor nutrition—two key factors that can affect how cancer behaves and how well a patient tolerates treatment [24].

These findings are in line with previous studies in various malignancies where mGPS has been shown to correlate independently with OS [25,26,27,28,29]. In ovarian cancer specifically, systematic reviews and meta-analyses have reported similar inverse associations between mGPS and survival outcomes [30].

Importantly, in our multivariate model, CRP and albumin as individual variables did not retain significance, suggesting that the mGPS—by integrating both inflammation and nutritional status—offers superior prognostic stratification. Similarly, age was not an independent predictor of OS, whereas ECOG performance status remained significant, emphasizing the relevance of functional status in prognostic assessment.

One of the most important strengths of mGPS lies in its clinical feasibility. It requires no specialized testing, incurs no additional cost, and can be calculated using routine blood tests that are already part of standard pre-treatment assessments. For clinicians, mGPS may serve as a practical tool to identify high-risk patients at diagnosis who could benefit from intensified monitoring, earlier intervention, or enrollment in clinical trials.

The clinical utility of mGPS lies in its simplicity and applicability. As a non-invasive, cost-effective, and easily reproducible tool, it can be seamlessly incorporated into routine pre-treatment evaluations to help identify high-risk patients who may benefit from more intensive surveillance or therapeutic strategies.

This study has several strengths, including the use of real-world data, consistent laboratory measurements, and a well-defined cohort of patients with FIGO stage III–IV disease. However, certain limitations should be acknowledged. The retrospective and single-center design may limit generalizability. The cohort size was modest, which may affect the statistical power. Additionally, potential confounding factors such as comorbid conditions or subclinical infections that might influence inflammatory markers were not fully accounted for. Lastly, although the sample size was relatively modest, the findings remained statistically robust.

## 5. Conclusions

The modified Glasgow Prognostic Score (mGPS) is a simple and cost-effective tool that independently predicts overall survival in advanced-stage epithelial ovarian cancer. By reflecting both inflammation and nutritional status, it enhances prognostic accuracy. Its integration with clinical factors like ECOG performance status may aid in personalized treatment decisions. Further validation in larger, prospective studies is recommended.

## Figures and Tables

**Table 1:** Clinicopathological characteristics of the study population.

Variable	*n* (%) or Value
Age (median [range], years)	56.1 (24.79–84.3)
ECOG performance status	
0–1	77 (86.5%)
2–3	12 (13.5%)
FIGO stage	
Stage III	61 (68.8%)
Stage IV	28 (31.2%)
Albumin (g/dL, mean ± SD)	3.6 ± 0.65
CRP (mg/L, mean ± SD)	21.86 ± 20.47
Modified Glasgow prognostic score	
Score 0	17 (19.1%)
Score 1	49 (55.1%)
Score 2	23 (25.8%)
Overall survival (months, median, range)	32.3 (178.4)

## Data Availability

The datasets generated and/or analyzed during the current study are available from the corresponding author upon reasonable request.
